# An Energy Efficient Mutual Authentication and Key Agreement Scheme Preserving Anonymity for Wireless Sensor Networks

**DOI:** 10.3390/s16060837

**Published:** 2016-06-08

**Authors:** Yanrong Lu, Lixiang Li, Haipeng Peng, Yixian Yang

**Affiliations:** 1 Information Security Center, State Key Laboratory of Networking and Switching Technology, Beijing University of Posts and Telecommunications, Beijing 100876, China; luyanrong1985@bupt.edu.cn (Y.L.); penghaipeng@bupt.edu.cn (H.P.); yxyang@bupt.edu.cn (Y.Y.); 2 National Engineering Laboratory for Disaster Backup and Recovery, Beijing University of Posts and Telecommunications, Beijing 100876, China

**Keywords:** anonymity, mutual authentication, wireless sensor networks, smart card

## Abstract

WSNs (Wireless sensor networks) are nowadays viewed as a vital portion of the IoTs (Internet of Things). Security is a significant issue in WSNs, especially in resource-constrained environments. AKA (Authentication and key agreement) enhances the security of WSNs against adversaries attempting to get sensitive sensor data. Various AKA schemes have been developed for verifying the legitimate users of a WSN. Firstly, we scrutinize Amin-Biswas’s currently scheme and demonstrate the major security loopholes in their works. Next, we propose a lightweight AKA scheme, using symmetric key cryptography based on smart card, which is resilient against all well known security attacks. Furthermore, we prove the scheme accomplishes mutual handshake and session key agreement property securely between the participates involved under BAN (Burrows, Abadi and Needham) logic. Moreover, formal security analysis and simulations are also conducted using AVISPA(Automated Validation of Internet Security Protocols and Applications) to show that our scheme is secure against active and passive attacks. Additionally, performance analysis shows that our proposed scheme is secure and efficient to apply for resource-constrained WSNs.

## 1. Introduction

With the advancement of short range radio communication coupled with advances in miniaturization of computing devices, WSNs (Wireless sensor networks) have drawn continuing attention from both academia and industrial areas due to its deployment scalability, power consumption constraint and wide applications. Within the infrastructure of WSNs, privacy and security are the two major challenges since nodes are generally deployed in hostile environments thus making the nodes vulnerable to attacks. From this context, secure information exchange over an untrusted network is a widely discussed issue in WSNs. In order to allow remote authorized users to access reliable sensor nodes which have been verified as legitimate ones, mutual AKA (Authentication and key agreement) between communicating entities is required in the scheme design. An AKA scheme for WSNs is composed of three classes of entity: users, sensor nodes and a gateway node (GWN), and has registration, login, authentication and key agreement, and password change phases. To date, research in an efficient and robust user authentication and session key agreement mechanism has gained a great deal of attention. A number of AKA schemes are developed in an attempt to enhance the security of the WSNs in the literature [1,2,3]. Among different kinds of cryptographic primitives (RSA [4], ECC [5,6] Elgamal [7] *etc.*) utilized in AKA for WSNs, lower computational cost scheme is even more admired owing to stringent constraints on limited computation capability, energy resources, storage and bandwidth of sensor nodes.

Wong *et al.* [8] released a hash function based AKA scheme for WSN, which sharply decreases computational load and makes the scheme adapt into a WSN environment. Nevertheless, as the scheme remains the lookup table of the registered user’s private data in the GWN side, it was demonstrated to be defenseless to stolen-verifier attack [9]. Later on, Das [9] developed a better scheme in order to mitigate the security flaws over Wong *et al*. The scheme concentrates on temporal credential and timestamp under defense mechanism aiming at preventing DoS attack efficiently while maintaining lightweight style. Unfortunately, the scheme was analyzed by many researchers and the results illustrated that it had still some drawbacks and flaws [10,11,12,13,14], such as incapability of achieving mutual authentication, notwithstanding node compromise attack, failing to provide the user password update securely. With the hope of amending aforementioned security weaknesses, several authors developed modifications on Das’s scheme but at the cost of increasing computational complexity [10,11,14]. Motivated by the thought of achieving better security and efficiency, Das *et al.*’s [15] built an efficient password based user AKA using only the hash function which encompasses the power of smart cards. They justified that compromise of a cluster head is free from node capture attacks. Their scheme allows only updating the password of the user locally without the help of the base station. Further, they evaluated their scheme in support of using no high computation except from the nominal task of assigning identity bits commitments and justified low memory requirement due to small size of identity bits commitment. Nevertheless, Turkanović [16], Wang-Wang [17] and Li [18] came across some additional problems in Das’s scheme, like non resistance to insider, stolen-verifier and node capture attacks. After that, Xue *et al.* [19] proposed a temporal-credential-based lightweight and resource user AKA scheme for WSNs using hash and XOR computations. In their scheme, the gateway node issues a temporal credential to each user and sensor node with the help of password-based authentication. Unfortunately, He *et al.* [20] was later remarked that the scheme of Xue *et al.* is imperfection and not applicable for practical implementation, due to some design defects and susceptibility to some attacks. Most recently, Turkanović *et al.* [21] proposed a lightweight user authentication scheme for WSN based only on hash and Xor computations that tend to save both computation and communication resources. Such cryptographic techniques scheme launched with a claim of achieving the basic security attributes as well as thwarting many attacks along with better complexities. The AKA scheme drew considerable attention but was subsequently on determined insecure and susceptible. The authors of [22,23,24] studied the vulnerability of the scheme [21] that incurs several security drawbacks and not applicable for practical implementation in the presence of an attacker who can mount a smart card theft attack. Motivated by the thought of preventing the security threats of scheme [21], Amin-Biswas [24] developed a modified version of the hash and Xor operations in order to appropriate for resource constrained environments. The authors addressed both security and efficiency, claimed that their designs possess many attractive features in which the system contains multiple gateway nodes. However, problems related to the leakage of the session short-term secrets accidentally are the fatal pitfalls of such scheme. Our contribution is motivated by the above facts.

## 2. Review of Amin-Biswas’s Scheme

**Figure 1:**
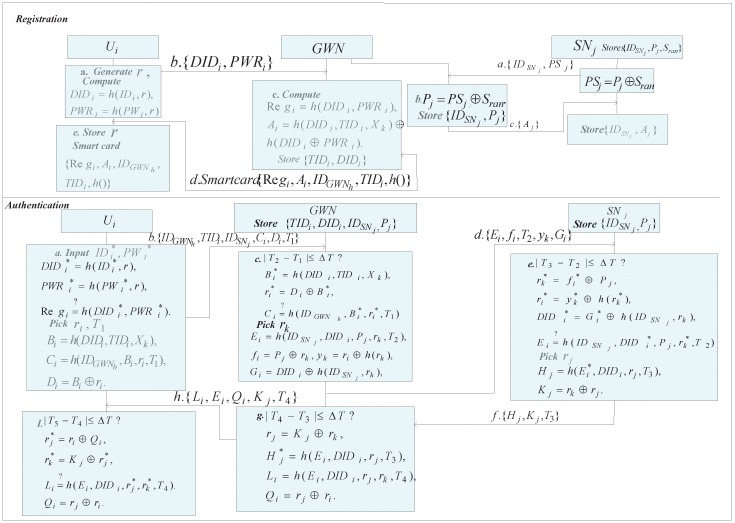
Mutual authentication and key agreement of Amin-Biswas’s scheme.

This section briefly reviews Amin-Biswas’s scheme, which consists of system setup phase, user and sensor node registration phases, login phase, authentication phase (Figure 1), password update phase and dynamic node addition phase. Moreover, their scheme is composed of three entities: user, gateway node, and sensor node. For convenience of description, Table 1 shows the notations used in Amin-Biswas’s scheme.

### 2.1. System Setup

The system administrator deploys each $SN_{j}$ which stores $\left\{ID_{SN_{j}},P_{j},S_{ran}\right\}$ into its memory, where $P_{j}=h\left(ID_{SN_{j}},S_{ran}\right)$, $S_{ran}$ is a random number and is known to all the GWNs and maintains it securely.

### 2.2. Sensor Node Registration

*Step 1*: $SN_{j}$ sends $\left\{ID_{SN_{j}},PS_{j}\right\}$ to the nearby GWN, where $PS_{j}=P_{j}⊕S_{ran}$.

*Step 2*: The GWN stores $\left\{ID_{SN_{j}},P_{j}\right\}$, where $P_{j}=PS_{j}⊕S_{ran}$. After that, the GWN sends a confirmation message to each sensor node.

*Step 3*: Upon receiving the confirmation message from the GWN, each $SN_{j}$ destroys $S_{ran}$ from the memory.

### 2.3. User Registration

*Step 1*: The new user $U_{i}$ computes $DID_{i}=h\left(ID_{i},r\right),PWR_{i}=h\left(PW_{i},r\right)$ and sends $\left\{DID_{i},PWR_{i}\right\}$ to the HGWN via private channel, where *r* is a nonce, $ID_{i}$ is the identity and $PW_{i}$ is the password of $U_{i}$.

*Step 2*: The HGWN computes $Reg_{i}=h\left(DID_{i},PWR_{i}\right),A_{i}=h\left(DID_{i},TID_{i},X_{k}\right)⊕h\left(DID_{i}⊕PWR_{i}\right)$, where $TID_{i}$ is a random identity and $X_{k}$ is the HGWN’s long term secret key.

*Step 3*: The HGWN issues a smart card which contains $\left\{Reg_{i},A_{i},ID_{GWN_{h}},TID_{i},h\left(\right)\right\}$ and sends it to $U_{i}$. Further, the HGWN stores $\left\{TID_{i},DID_{i}\right\}$ in its memory.

*Step 4*: When receiving the smart card, $U_{i}$ stores {r} in the smart card.

### 2.4. Login and Authentication

*Step 1*: $U_{i}$ inserts the smart card and inputs identity $ID_{i}$ and password $PW_{i}$ to the card reader. After that, the card reader computes $DID_{i}=h\left(ID_{i},r\right),PWR_{i}=h\left(PW_{i},r\right)$ and checks whether $h\left(DID_{i},PWR_{i}\right)\overset{?}{=}Reg_{i}$.

*Step 2*: If it matches, the card reader computes $B_{i}=h\left(DID_{i},TID_{i},X_{k}\right)=A_{i}⊕h\left(DID_{i}⊕PWR_{i}\right),$$C_{i}=h\left(ID_{GWN_{h}},B_{i},r_{i},T_{1}\right),D_{i}=B_{i}⊕r_{i}$ and sends a login message $M_{1}=\left\{ID_{GWN_{h}},TID_{i},ID_{SN_{j}},C_{i},D_{i},T_{1}\right\}$ to the HGWN by public channel.

*Step 3*: When receiving the message $M_{1}$, the HGWN first checks whether the received timestamp $T_{1}$ is within the valid time period, the HGWN computes $B_{i}=h\left(DID_{i},TID_{i},X_{k}\right),r_{i}=D_{i}⊕B_{i}$, the HGWN extracts $DID_{i}$ from the database using $TID_{i}$. Next, the HGWN checks whether $h\left(ID_{GWN_{h}},B_{i},r_{i},T_{1}\right)\overset{?}{=}C_{i}$. If it holds, the HGWN computes $E_{i}=h\left(ID_{SN_{j}},DID_{i},P_{j},r_{k},T_{2}\right),f_{i}=P_{j}⊕r_{k},y_{k}=r_{i}⊕h\left(r_{k}\right),G_{i}=DID_{i}⊕h\left(ID_{SN_{j}},r_{k}\right)$ and sends $M_{2}=\left\{E_{i},f_{i},G_{i},y_{k},T_{2}\right\}$ to the the sensor node $SN_{j}$ via public channel.

*Step 4*: After receiving the message $M_{2}$, $SN_{j}$ checks whether $|T_{3}-T_{2}|\leq \Delta T$. If it holds, $SN_{j}$ computes $r_{k}=f_{i}⊕P_{j},r_{i}=y_{k}⊕h\left(r_{k}\right),DID_{i}=G_{i}⊕h\left(ID_{SN_{j}},r_{k}\right)$ and checks whether $h\left(ID_{SN_{j}},DID_{i},P_{j},r_{k},T_{2}\right)\overset{?}{=}E_{i}$. If it matches, $SN_{j}$ computes $H_{j}=h\left(E_{i},DID_{i},r_{j},T_{3}\right),K_{j}=r_{k}⊕r_{j}$ and sends $M_{3}=\left\{H_{j},K_{j},T_{3}\right\}$ to the HGWN via public channel.

*Step 5*: Upon receiving the message $M_{3}$, the HGWN first checks the timestamp validity, *i.e.*, $|T_{4}-T_{3}|\leq T$, where $T_{4}$ is the current timestamp. The HGWN computes $r_{j}=K_{j}⊕r_{k}$, $H_{j}=h\left(E_{i},DID_{i},r_{j},T_{3}\right)$. If it is true, the HGWN computes $L_{i}=h\left(E_{i},DID_{i},r_{j},r_{k},T_{4}\right),Q_{i}=r_{j}⊕r_{i}$ and sends $M_{4}=\left\{L_{i},E_{i},Q_{i},K_{j},T_{4}\right\}$ to the $U_{i}$ via public channel.

*Step 6*: After receiving the message $M_{4}$, $U_{i}$ checks whether the received timestamp is within the valid time intervals. If it holds, $U_{i}$ extracts $r_{j}=r_{i}⊕Q_{i},r_{k}=K_{j}⊕r_{j},L_{i}=h\left(E_{i},DID_{i},r_{j},r_{k},T_{4}\right)$. If it is true, $U_{i}$ confirms the authenticity of $SN_{j}$ and computes $SK=h(DID_{i},r_{i},r_{j},r_{k})$ between the entities involved in the system.

### 2.5. Dynamic Node Addition

According to the system setup phase, the system administrator deploys the new sensor node over the target region and the deployed sensor node executes sensor node registration phase to the nearby GWN.

### 2.6. Password Update

*Step 1*: A user keys his password $PW_{i}$, the card reader computes $\alpha_{i}=h\left(DID_{i},TID_{i},X_{k}\right)=A_{i}⊕h\left(DID_{i}⊕PWR_{i}\right)$ and then computes $PWR_{i}^{new}=h\left(PW_{i},r\right),Reg_{i}^{new}=h\left(DID_{i},PWR_{i}^{new}\right),A_{i}^{new}=\alpha_{i}⊕h\left(DID_{i}⊕PWR_{i}^{new}\right)$.

*Step 2*: The card reader stores the new computed values $\left\{Reg_{i}^{new},A_{i}^{new}\right\}$ instead of the old values $\left\{Reg_{i},A_{i}\right\}$.

## 3. Security Analysis of Amin-Biswas’s Scheme

Although Amin-Biswas claimed that their scheme achieves several security requirements including mutual authentication, user anonymity and resilience against some attacks. Unfortunately, we found that there was still something security vulnerability in Amin-Biswas’s scheme.

### Known Session-Specific Temporary Information Attack

**Figure 2:**
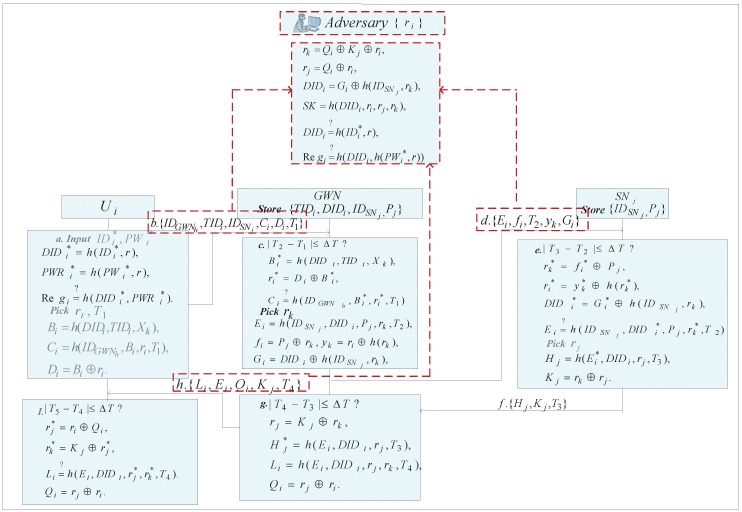
Known session-specific temporary information attack on Amin-Biswas’s schem.

Cheng *et al.* [25] has demonstrated that the exposure of session temporary information accidentally should not compromise the secrecy of generated session key. However, we will demonstrate that Amin-Biswas’s scheme contraries to this security property which is necessary for a good or an ideal authentication scheme [26]. Without loss of generality, we assume that a temporary information $r_{i}$ is compromised by an adversary unintentionally, which may allow the adversary to frame the session key effortlessly and even more acquire the legitimate user’s sensitive data by means of monitoring the transmitted data in the communication. To illustrate the process concretely, you can look at an attack in the next few steps (Figure 2).

*Step 1*: The adversary could extract the session ephemeral secrets $r_{k}$ and $r_{j}$ from the results of $Q_{i}⊕K_{j}⊕r_{i}$ and $Q_{i}⊕r_{i}$, where $Q_{i},K_{j}$ are the known parameters stemmed from the transferred message $M_{4}:\left\{L_{i},E_{i},Q_{i},K_{j},T_{4}\right\}$.

*Step 2*: Based on the derived the session immediate secret $r_{k}$, the adversary has ability to retrieve another important parameter $DID_{i}$ by computing $G_{i}⊕h\left(ID_{SN_{j}},r_{k}\right)$, where $G_{i}$ is also obtained through the transmitted messages $M_{1}:\left\{ID_{GWN_{h}},TID_{i},ID_{SN_{j}},C_{i},D_{i},T_{1}\right\}$ and $M_{2}:\left\{E_{i},f_{i},G_{i},y_{k},T_{2}\right\}$.

*Step 3*: The adversary could compute the session key $SK=h(DID_{i},r_{i},r_{j},r_{k})$ with all those derived data. Not only that, the adversary could easily guess the user’s identity $ID_{i}$ by attempting to check whether $DID_{i}\overset{?}{=}h\left(ID_{i}^{*},r\right)$ until making the equation true, where $ID_{i}^{*}$ is a candidate identity and *r* is extracted with a stolen smart card. The adversary is further capable of retrieving the user’s password $PW_{i}$ on the strength of the extracted secrets $\left\{Reg_{i}\right\}$ by checking $Reg_{i}\overset{?}{=}h\left(DID_{i},h\left(PW_{i}^{*},r\right)\right)$ from the legal user’s smart card. The aforementioned cryptanalysis is based on the concrete fact that identity and passwords are low-entropy keys [27,28]. As a result, the adversary succeeds to get the user’s identity $ID_{i}$ and the user’s password $PW_{i}$.

*Step 4*: The above analysis reveals that, all those information leaks allow the adversary to impersonate as a legitimate user to login the GWN and access the real-time information from sensor nodes. In other words, our analysis demonstrates that their scheme can be free from known session-specific temporary information attack, thereby Amin-Biswas’s scheme is completely insecure.

## 4. Proposed Improved Scheme

**Figure 3:**
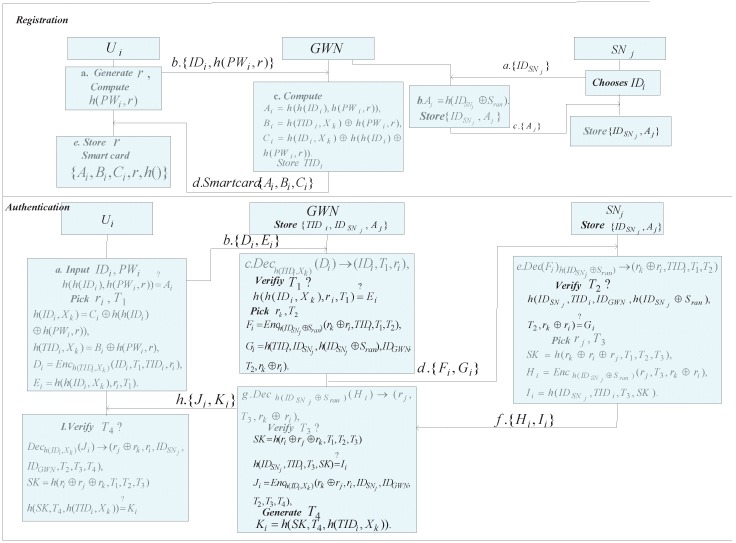
Mutual authentication and key agreement of our scheme.

This section will describe our proposed anonymity-preserving AKA scheme in detail. The proposed AKA scheme conceals the user’s real identity in the encryption algorithm along with the hash of random identity and secret key as the symmetric key. The messages, which are transmitted in public channel, are the results of the hash or the encryption, thus avoiding the risk by intercepting the communication channel to acquire the plaintext directly. In order to conquer the known session-specific temporary information attack, each communicate entity only knows the xor results of the others’ generated random numbers in computing the session key. The proposed AKA scheme inherits Amin-Biswas’s scheme aiming at cope with the loopholes of the aforementioned security drawbacks of their scheme. Based on the previous analysis, the functionality of the proposed scheme has been greatly improved with a slight higher computation cost due to the symmetric cryptographic algorithm. Our proposed AKA scheme has five phases: User registration; Sensor node registration; Login; Authentication and key agreement (Figure 3); Password change. We will introduce them as follows.

### 4.1. User Registration

*Step 1*: A new user $U_{i}$ chooses his identity $ID_{i}$ and password $PW_{i}$, then he sends his registration request message $\left\{ID_{i},h\left(PW_{i},r\right)\right\}$ to the gateway node GWN, where *r* is a random number.

*Step 2*: Upon receipt of the message, GWN computes $A_{i}=h\left(h\left(ID_{i}\right),h\left(PW_{i},r\right)\right),B_{i}=h\left(TID_{i},X_{k}\right)⊕h\left(PW_{i},r\right),C_{i}=h\left(ID_{i},X_{k}\right)⊕h\left(h\left(ID_{i}\right)⊕h\left(PW_{i},r\right)\right)$. Next, GWN issues a smart card for each user after storing $\left\{A_{i},B_{i},C_{i}\right\}$ into the memory of smart card and thus sends back it to $U_{i}$. At last, GWN stores $\left\{TID_{i}\right\}$ in its memory.

*Step 3*: After receiving the smart card, $U_{i}$ adds *r* to the smart card.

### 4.2. Sensor Node Registration

*Step 1*: The sensor node $SN_{j}$ transmits its identity $ID_{SN_{j}}$ to GWN.

*Step 2*: GWN computes $A_{j}=h\left(ID_{SN_{j}}⊕S_{ran}\right)$ and returns it to $SN_{j}$ after storing $\left\{ID_{SN_{j}},A_{j}\right\}$ into its memory.

*Step 3*: When receiving the message from GWN, $SN_{j}$ also keeps them securely.

### 4.3. Login

When a registered user $U_{i}$ desires the WSNs services, he needs to be prepare his personal information along with the smart card. The following procedure are required to be done by $U_{i}$: 

*Step 1*: $U_{i}$ enters his identity $ID_{i}$ and password $PW_{i}$ into the smart card after inserting the smart card into the mobile device. The smart card computes $h(h\left(ID_{i}\right),h\left(PW_{i},r\right))$ and checks whether it is equal to $A_{i}$. If it holds, $U_{i}$ is considered as a legal user.

*Step 2*: The card reader derives $h(TID_{i},X_{k})$ and $h(ID_{i},X_{k})$ by computing $B_{i}⊕h\left(PW_{i},r\right)$ and $C_{i}⊕h\left(h\left(ID_{i}\right)⊕h\left(PW_{i},r\right)\right)$, respectively. Based on the two values, the card reader computes $D_{i}$ by encrypting the information $\left\{ID_{i},T_{1},TID_{i},r_{i}\right\}$ with the derived $h(TID_{i},X_{k})$ and computes $E_{i}$ by putting the information $\left\{h\left(ID_{i},X_{k}\right),r_{i},T_{1}\right\}$ into the hash function, where $T_{1}$ is the current timestamp at user side and $r_{i}$ is a random number. Next, the card reader sends a login message $\left\{D_{i},E_{i}\right\}$ to GWN.

*Step 3*: Upon receiving the login message, GWN decrypts $D_{i}$ by the symmetric key $h(TID_{i},X_{k})$ to retrieve $\left\{ID_{i},T_{1},r_{i}\right\}$. Next, GWN checks whether $|T_{2}-T_{1}|\leq \Delta T$, where $T_{2}$ is the current timestamp at GWN side. If it is valid, GWN verifies $h\left(h\left(ID_{i},X_{k}\right),r_{i},T_{1}\right)\overset{?}{=}E_{i}$. The validation of $E_{i}$ ensures $U_{i}$ is a legitimate user. Subsequently, GWN picks a random number $r_{k}$ and computes $F_{i}=Enc_{h(ID_{SN_{j}}⊕S_{ran})}\left(r_{k}⊕r_{i},TID_{i},T_{1},T_{2}\right)$, $G_{i}=h\left(TID_{i},ID_{SN_{j}},h\left(ID_{SN_{j}}⊕S_{ran}\right),ID_{GWN},T_{2},r_{k}⊕r_{i}\right)$. Next, GWN sends the message $\left\{F_{i},G_{i}\right\}$ to $SN_{j}$.

*Step 4*: When receiving the message from GWN, $SN_{j}$ decrypts $F_{i}$ using the symmetric key $h(ID_{SN_{j}}⊕S_{ran})$ to derive $\left\{r_{k}⊕r_{i},TID_{i},T_{1},T_{2}\right\}$. And then, $SN_{j}$ checks the timestamp $T_{2}$ is within a permissible temporal interval. Next, $SN_{j}$ computes $h(ID_{SN_{j}},TID_{i},ID_{GWN},h\left(ID_{SN_{j}}⊕S_{ran}\right),T_{2},r_{k}⊕r_{i})$ and checks whether it matches with the received $G_{i}$. It it holds, $SN_{j}$ computes $SK=h(r_{k}⊕r_{i}⊕r_{j},T_{1},T_{2},T_{3})$, $H_{i}=Enc_{h(ID_{SN_{j}}⊕S_{ran})}\left(r_{j},T_{3},r_{k}⊕r_{i}\right)$, $I_{i}=h\left(ID_{SN_{j}},TID_{i},T_{3},SK\right)$. Finally, $SN_{j}$ transmits the message $\left\{H_{i},I_{i}\right\}$ to GWN.

*Step 5*: After receiving the message from $SN_{j}$, GWN also needs to decrypt the received $H_{i}$ to derive $\left\{r_{j},T_{3},r_{k}⊕r_{i}\right\}$. Upon retrieving $T_{3}$, GWN verifies whether $T_{3}$ is a valid timestamp. If it is valid, GWN computes $SK=h(r_{k}⊕r_{i}⊕r_{j},T_{1},T_{2},T_{3})$ and checks whether $h\left(ID_{SN_{j}},TID_{i},T_{3},SK\right)\overset{?}{=}I_{i}$. If it is correct, GWN computes $J_{i}=Enc_{h(ID_{i},X_{k})}\left(r_{k}⊕r_{j},r_{i},ID_{SN_{j}},ID_{GWN},T_{2},T_{3},T_{4}\right)$ and $K_{i}=h\left(SK,T_{4},h\left(TID_{i},X_{k}\right)\right)$, where $T_{4}$ is the current timestamp at GWN side. Next, GWN sends the message $\left\{J_{i},K_{i}\right\}$ to $U_{i}$.

*Step 6*: Once receiving the message from GWN, $U_{i}$ derives $\left\{r_{j}⊕r_{k},ID_{SN_{j}},ID_{GWN},T_{2},T_{3},T_{4}\right\}$ by decrypting $J_{i}$ using the symmetric key $h(ID_{i},X_{k})$. $U_{i}$ then checks whether $T_{4}$ is fresh. The freshness of $T_{4}$ is verified, $U_{i}$ proceeds to compute the session key $SK=h(r_{k}⊕r_{i}⊕r_{j},T_{1},T_{2},T_{3})$ and examine whether $h(SK,T_{4},h\left(TID_{i},X_{k}\right))$ is equivalent to the received $K_{i}$. If the equation is true, the handshake among three-party is successful, and they negotiate the session key SK with each other. The establishment of the session key is considered to be encrypted the following packs in their communication channel.

### 4.4. Password Change

When a user attempts to update his password into a new one, he needs to execute the following steps: 

*Step 1*: The user initially inserts the smart card into the card reader and inputs his identity $ID_{i}$ and old password $PW_{i}$. Next, the card reader computes $h(h\left(ID_{i}\right),h\left(PW_{i},r\right))$ and checks whether it is equal to $A_{i}$. If it holds, the user is considered as a legal one. And thus, the card reader asks the user to key a new password.

*Step 2*: After keying the new password, the card reader computes $A_{i}^{*}=h\left(h\left(ID_{i}\right),h\left(PW_{i},r\right)\right)$, $B_{i}^{*}=B_{i}⊕h\left(PW_{i},r\right)⊕h\left(PW_{i}^{*},r\right)$ and $C_{i}^{*}=C_{i}⊕h\left(h\left(ID_{i}\right)⊕h\left(PW_{i},r\right)\right)⊕h\left(h\left(ID_{i}\right)⊕h\left(PW_{i}^{*},r\right)\right)$. The card reader replaces $\left\{A_{i},B_{i},C_{i}\right\}$ with $\left\{A_{i}^{*},B_{i}^{*},C_{i}^{*}\right\}$.

## 5. Security Analysis of Our Scheme

In this section, the strength of the proposed AKA scheme by considering the informal and formal analysis has been analyzed. To be specific, our scheme keeps to the system requirements and successfully withstands diverse attacks to enhance the security level. Next, using BAN logic [29] to demonstrate the validity of our AKA scheme. Then, the formal security analysis of our scheme is presented. Besides, the widely-accepted AVISPA tool [28,29] is used to simulated for the security experimental verification of our AKA scheme.

### 5.1. Informal Security Analysis

This section addresses a detailed security evaluation to indicate that the proposed scheme is secure against various known security attacks. Suppose that an adversary A can eavesdrop, intercept, modify, delete or replay the transmission over a public channel.

#### 5.1.1. Session Key Agreement

The session key is established among the user $U_{i}$, the sensor node $SN_{j}$ and the gate-way node GWN. Note that $U_{i}$ and $SN_{j}$ has no way to know other participates’ random numbers excepts themselves. The established session key is to encrypt the real-time data to ensure the transmission are confidential through an unreliable channel. Therefore, the session key is different in each session due to it is generated by various random numbers, and it is challenging for A to extract the current session key from the eavesdropped messages because of the one-way property of the hash function.

#### 5.1.2. Mutual Authentication

The gate-way node GWN first checks whether the received timestamp $T_{1}$ is valid as compare to the decrypted one from $D_{i}$ when receiving the message $\left\{D_{i},E_{i},T_{1}\right\}$. Next, GWN verifies $h\left(h\left(ID_{i},X_{k}\right),r_{i},T_{1}\right)\overset{?}{=}E_{i}$. If both the condition are true, the validity of the user $U_{i}$ is authenticated by GWN. Similarly, $U_{i}$ checks the validness of the received timestamp with the derived one from $J_{i}$ after receiving the message $\left\{J_{i},K_{i},T_{4}\right\}$. He then checks whether $h\left(SK,T_{4},T_{1},T_{2}\right)\overset{?}{=}K_{i}$. If both the equation hold, the validity of GWN is confirmed by $U_{i}$ and thus the sensor node $SN_{j}$ is also verified due to only the valid $SN_{j}$ would forward the correct random number $r_{j}$ and thus compute the correct session key. Correspondingly, mutual authentication between $SN_{j}$ and GWN are performed by checking $G_{i}$ and $I_{i}$. With the same verification mode as GWN and $U_{i}$, double authentication is utilized, *i.e.*, to verify the freshness of the received timestamp with the retrieved one, to put the retrieved one to substitute in the awaiting verification value and thus checking the hashed value. In this way, A has no ability to modify the hashed value and only modify the timestamp, thus impersonating as any participates. Therefore, mutual authentication among the entities are provided in the proposed scheme.

#### 5.1.3. Resistance to Insider Attack

It is probable that the users use the same identity and password across multiple networks. In our case, the GWN plays the role of a trusted third party, but some curious administrator can have access to the database which stores the user’s personal information in order to gain something important. However, during the registration phase, the user $U_{i}$ transmitted masked password $h(PW_{i},r)$ instead of plaintext password. In this way, the insider of system has no ability to derive the privacy of the user because of non-invertible property of one-way hash function. Therefore, the proposed AKA scheme is resilient against the privileged insider attack.

#### 5.1.4. User Anonymity

We adopt two strategies to protect the user’s identity from disclosing. One is the masked identity $h(ID_{i},X_{k})$ with the secret key $X_{k}$ of GWN. Note that the key is essentially a random number generated by GWN and thus it is computationally infeasible for A to extract the user’s identity in plaintext. Another is directly the use of dynamic identity selected by GWN, which is hashed in the open channel. In essence, the random identity is no relation with the real one. Consequently, compromise of released one influences nothing on the actual identity of $U_{i}$. Therefore, the proposed scheme mechanism is a dynamic identification process and we will verify the point later in simulation.

#### 5.1.5. Resistance to Known Session-Specific Temporary Information Attack

Known session-specific temporary information security means if A gets the ephemeral information, such as the random values, $r_{a}\left(a=i,k,j\right)$ and $X_{k}$, he still cannot acquire information of the session key. Since A has no way to compute the symmetrical key $h(ID_{i},X_{k})$ without knowing the identity of $ID_{i}$ and thus decrypting the packs transmitted in communication channel. More seriously, $U_{i}$ and $SN_{j}$ only receive the results of xor for the random numbers picked by the rest of participates. As such, attempting to intercept any hashed values in the public communication channel but are unhelpful to compute the session key. Therefore, it is not possible for any attacker to compute the session key on leakage or compromise of session specific temporary information.

#### 5.1.6. Resistance to Denial-of-Service Attack

This attack is to secure against since our proposed scheme works on the principle of request-response communication. Additionally, the sensor node $SN_{j}$ will check the received packs and chooses refuse or pass the session from the sender. On the other hand, if A does the malicious flooding of the authentication requests to $SN_{j}$, GWN first knows about malicious dropping of such control messages as a referee. And A needs to know the symmetric key between the legal user and the legitimate sensor node unless he can solve the one-way hash functions. Furthermore, we have introduced timestamps into the scheme, which mitigate any consequential request. As such, we say that our scheme has also the ability to withstand the denial-of-service attack.

#### 5.1.7. Resistance to Sensor Node Impersonation Attack

Suppose A gets all transmitted information such as $\left\{E_{i},F_{i},G_{i}\right\}$ and $\left\{H_{i},I_{i}\right\}$ and plans to impersonate as a legitimate sensor node. However, it has no feasible way to decrypt the cryptographic packs like $F_{i}$ without knowing the symmetry key with the GWN, thus failing to compute the correct session key and thus excluding by GWN. Therefore, A can not impersonate as a valid sensor node.

#### 5.1.8. Resistance to Off-Line Password Guessing with Smart Card Breach Attack

The system is secure even if the stored information $\left\{A_{i},B_{i},C_{i},r,h\left(\right)\right\}$ and the login message $\left\{D_{i},E_{i}\right\}$ are revealed. Since the user’s identity and password are hashed by GWN’s long-term private $X_{k}$. The adversary A has no information about these private keys. Therefore, the proposed scheme is secure against off-line password guessing attack.

### 5.2. Authentication Proof Based on the BAN Logic

The BAN logic, which is the first suggestion to formalize the description and analysis of authentication schemes, is used to analyze existing schemes to bring out their flaws. We analyze the proposed scheme by establishing some required goals, making some assumptions about the initial state of the scheme and transforming the proposed AKA scheme to the idealized form. Some descriptions about its notations and formulas are shown as follows.

  ***Notations* & *Formulas***

  ·: PX: *P* has received message *X*

·: P|≡X: *P* believes *X*

·: P|∼X: *P* once said *X*

·: P⇒X: *P* has jurisdiction over *X*

·: $P\overset{K}{\to }Q$: *P* and *Q* shared key *K*

·: #(X): *X* is fresh

·: $<X{>}_{K}$: the formula *X* encrypted under the formula *K*

·: (X,Y): *X* or *Y* is one part of (X,Y)

·: $P\overset{K}{\Leftrightarrow }Q$: *P* and *Q* share secret *K*

  ·: Message meaning rule: $\frac{P|\equiv P\overset{K}{↔}Q,\;P◃{\left\{X\right\}}_{K}}{P|\equiv Q|∼X}$

  ·: Nonce-verification rule: $\frac{P|\equiv \#(X),\;P|\equiv Q|∼X}{P|\equiv Q|\equiv X}$

  ·: Jurisdiction rule: $\frac{P|\equiv Q\Rightarrow X,\;P|\equiv Q|\equiv X}{P|\equiv X}$

  ·: Belief rule: $\frac{P|\equiv Q|\equiv (X,Y)}{P|\equiv Q|\equiv X}$

  ·: Freshness distribution rule: $\frac{P|\equiv \#X}{P|\equiv \#(X,Y)}$

  ***Aims***

  $Aim_{1}$. $GWN|\equiv ID_{i}$

  $Aim_{2}$. $SN_{j}|\equiv SN_{j}\overset{SK}{⇌}GWN$

  $Aim_{3}$. $SN_{j}\left|\equiv GWN\right|\equiv SN_{j}\overset{SK}{⇌}GWN$

  $Aim_{4}$. $GWN|\equiv SN_{j}\overset{SK}{⇌}GWN$, $GWN|\equiv U_{i}\overset{SK}{⇌}GWN$

  $Aim_{5}$. $GWN|\equiv U_{i}|\equiv U_{i}\overset{SK}{⇌}GWN$

  $Aim_{6}$. $GWN|\equiv SN_{j}|\equiv SN_{j}\overset{SK}{⇌}GWN$

  $Aim_{7}$. $U_{i}|\equiv U_{i}\overset{SK}{⇌}GWN$

  $Aim_{8}$. $U_{i}\left|\equiv GWN\right|\equiv U_{i}\overset{SK}{⇌}GWN$

  $Aim_{9}$. $SN_{j}\left|\equiv U_{i}\right|\equiv U_{i}\overset{SK}{⇌}S_{j}$

  $Aim_{10}$. $U_{i}\left|\equiv SN_{j}\right|\equiv U_{i}\overset{SK}{⇌}S_{j}$

  ***Idealization***

  $U_{i}\to GWN$: $\left\{D_{i},E_{i}\right\}$

  $D_{i}$: $<ID_{i},T_{1},TID_{i},r_{i}{>}_{U_{i}\overset{h(TID_{i},X_{k})}{⇌}GWN}$, $E_{i}$: $<h\left(ID_{i},X_{k}\right),r_{i},T_{1}>$

  $GWN\to SN_{j}$: $\left\{TID_{i},F_{i},G_{i},T_{2}\right\}$

  $F_{i}$: $<r_{k}⊕r_{i},ID_{SN_{j}},T_{1},T_{2}{>}_{GWN\overset{h(ID_{SN_{j}}⊕X_{k})}{⇌}SN_{j}}$, $G_{i}$: $<ID_{SN_{j}},TID_{i},ID_{GWN},T_{2},r_{k}⊕r_{i}{>}_{GWN\overset{h(ID_{SN_{j}}⊕X_{k})}{⇌}SN_{j}}$

  $SN_{j}\to GWN$: $\left\{H_{i},I_{i},T_{3}\right\}$

  $H_{i}$: $<r_{j},T_{3},r_{i}⊕r_{k}{>}_{GWN\overset{h(ID_{SN_{j}}⊕X_{k})}{⇌}SN_{j}}$, $I_{i}$: $<ID_{SN_{j}},TID_{i},T_{3},T_{2},SK{>}_{GWN\overset{SK}{⇌}SN_{j}}$

  $GWN\to U_{i}$: $\left\{J_{i},K_{i},T_{4}\right\}$

  $J_{i}$: $<r_{k}⊕r_{j},r_{i},ID_{{SN}_{j}},ID_{GWN},T_{2},T_{3},T_{4}{>}_{U_{i}\overset{h(TID_{i},X_{k})}{⇌}GWN}$

  $K_{i}$: $<SK,T_{4},h\left(TID_{i},X_{k}\right){>}_{GWN\overset{SK}{⇌}U_{i}}$

  ***Assumptions***

  $A_{1}$: $U_{i}|\equiv \#r_{i}$

  $A_{2}$: $GWN|\equiv U_{i}\overset{h(TID_{i},X_{k})}{⇌}HGWN$

  $A_{3}$: $U_{i}|\equiv U_{i}\overset{h(TID_{i},X_{k})}{⇌}HGWN$

  $A_{4}$: $GWN|\equiv GWN\overset{h(ID_{SN_{j}},X_{k})}{⇌}SN_{j}$

  $A_{5}$: $SN_{j}|\equiv GWN\overset{h(ID_{SN_{j}},X_{k})}{⇌}SN_{j}$

  $A_{6}$: $GWN|\equiv \#TID_{i}$

  $A_{7}$: $GWN|\equiv U_{i}\Rightarrow ID_{i}$

  $A_{8}$: $GWN|\equiv X_{k}$

  $A_{9}$: $SN_{j}|\equiv ID_{SN_{j}}$

  $A_{10}$: $GWN|\equiv U_{i}\Rightarrow r_{i}$

  $A_{11}$: $SN_{j}|\equiv GWN\Rightarrow r_{k}$

  $A_{12}$: $SN_{j}|\equiv \#\left(r_{i},r_{k},r_{j}\right)$

  $A_{13}$: $GWN|\equiv \#(r_{i},r_{k},r_{j})$

  $A_{14}$: $GWN|\equiv SN_{j}\Rightarrow r_{j}$

  $A_{15}$: $U_{i}|\equiv U_{i}\overset{h(ID_{i},X_{k})}{⇌}GWN$

  ***Derivation process***

  According to $D_{i}$, we get:

  $D_{1}$. $GWN◃<ID_{i},T_{1},TID_{i},r_{i}{>}_{U_{i}\overset{h(TID_{i},X_{k})}{⇌}GWN}$

  According to $D_{1}$, $A_{2}$ and message rule, we derive:

  $D_{2}$. $GWN|\equiv U_{i}∼\left(ID_{i},T_{1},TID_{i},r_{i}\right)$

  According to $A_{6}$, $D_{2}$ and freshness distribution rule, we gain:

  $D_{3}$. $GWN|\equiv \#(ID_{i},T_{1},TID_{i},r_{i})$

  According to $D_{2}$-$D_{3}$ and nonce-verification rule, we achieve:

  $D_{4}$. $GWN|\equiv U_{i}|\equiv \left(ID_{i},T_{1},TID_{i},r_{i}\right)$

  According to $D_{4}$ and belief rule, we acquire:

  $D_{5}$. $GWN|\equiv U_{i}|\equiv ID_{i},GWN|\equiv U_{i}|\equiv r_{1},GWN\left|\equiv U_{i}\right|\equiv T_{1}$

  According to $D_{5}$, $A_{7}$ and jurisdiction rule, we attain:

  $D_{6}$. $GWN|\equiv ID_{i}\left(Aim_{1}\right)$, $GWN|\equiv r_{i},GWN|\equiv T_{1}$

  According to $Aim_{1}$, $A_{8}$ and jurisdiction rule, we get:

  $D_{7}$. $GWN|\equiv h(ID_{i},X_{k})$

  According to $F_{i}$, we collect:

  $D_{8}$. $SN_{j}◃<r_{k}⊕r_{i},ID_{SN_{j}},T_{1},T_{2}{>}_{GWN\overset{h(ID_{SN_{j}}⊕X_{k})}{⇌}SN_{j}}$

  According to $D_{8}$, $A_{5}$ and message rule, we seek:

  $D_{9}$. $SN_{j}|\equiv GWN∼\left(r_{k}⊕r_{i},ID_{SN_{j}},T_{1},T_{2}\right)$

  According to $A_{9}$ and freshness distribution rule, we receive:

  $D_{10}$. $SN_{j}|\equiv \#\left(r_{k}⊕r_{i},ID_{SN_{j}},T_{1},T_{2}\right)$

  According to $D_{9}$-$D_{10}$ and nonce-verification rule, we extract:

  $D_{11}$. $SN_{j}\left|\equiv GWN\right|\equiv \left(r_{k}⊕r_{i},ID_{SN_{j}},T_{1},T_{2}\right)$

  According to $A_{10}$-$A_{11}$, $D_{5}$ and jurisdiction rule, we derive:

  $D_{12}$. $SN_{j}|\equiv GWN\Rightarrow r_{k}⊕r_{i}$

  According to $D_{11}$-$D_{12}$, and jurisdiction rule, we regain:

  $D_{13}$. $SN_{j}|\equiv r_{k}⊕r_{i}$

  According to $D_{13}$, $A_{12}$ and $SK=h(r_{k}⊕r_{i}⊕r_{k})$

  $Aim_{2}$. $SN_{j}|\equiv SN_{j}\overset{SK}{⇌}GWN$

  According to $Aim_{2}$, $A_{12}$ and nonce verification rule, we earn:

  $Aim_{3}$. $SN_{j}\left|\equiv GWN\right|\equiv SN_{j}\overset{SK}{⇌}GWN$

  According to $H_{i}$, we get:

  $D_{14}$. $GWN◃<r_{j},T_{3},r_{i}⊕r_{k}{>}_{GWN\overset{h(ID_{SN_{j}}⊕X_{k})}{⇌}SN_{j}}$

  According to $D_{14}$, $A_{4}$ and message rule, we seek:

  $D_{15}$. $GWN|\equiv SN_{j}|∼\left(r_{j},T_{3},r_{i}⊕r_{k}\right)$

  According to $D_{15}$, $A_{13}$, $D_{6}$ and freshness distribution rule, we gain:

  $D_{16}$. $GWN|\equiv SN_{j}|\equiv \#\left(r_{j},T_{3},r_{i}⊕r_{k}\right)$

  According to $D_{15}$-$D_{15}$ and nonce-verification rule, we derive:

  $D_{17}$. $GWN|\equiv SN_{j}|\equiv \left(r_{j},T_{3},r_{i}⊕r_{k}\right)$

  According to $D_{17}$ and belief rule, we get:

  $D_{18}$. $GWN|\equiv SN_{j}|\equiv r_{j}$

  According to $D_{18}$, $A_{14}$ and jurisdiction rule, we regain:

  $D_{19}$. $GWN|\equiv r_{j}$

  According to $D_{19}$, $A_{13}$, $D_{6}$ and $SK=h(r_{j}⊕r_{i}⊕r_{k})$

  $Aim_{4}$. $GWN|\equiv U_{i}\overset{SK}{⇌}GWN$, $GWN|\equiv SN_{j}\overset{SK}{⇌}GWN$

  According to $Aim_{4}$, $A_{13}$ and nonce-verification rule, we collect:

  $Aim_{5}$. $GWN|\equiv U_{i}|\equiv U_{i}\overset{SK}{⇌}GWN$

  According to $I_{i}$, we obtain:

  $D_{20}$. $GWN◃<ID_{SN_{j}},TID_{i},T_{3},T_{2},SK{>}_{GWN\overset{SK}{⇌}SN_{j}}$

  According to $Aim_{2}$, $Aim_{4}$, $D_{20}$ and message meaning rule, we get:

  $D_{21}$. $GWN|\equiv SN_{j}|∼\left(ID_{SN_{j}},TID_{i},T_{3},T_{2},SK\right)$

  According to $D_{21}$, $Aim_{4}$ and nonce-verification rule, we regain:

  $Aim_{6}$. $GWN|\equiv SN_{j}|\equiv SN_{j}\overset{SK}{⇌}GWN$

  According to $J_{i}$, we attain:

  $D_{22}$. $U_{i}◃<r_{k}⊕r_{j},r_{i},ID_{{SN}_{j}},ID_{GWN},T_{2},T_{3},T_{4}{>}_{U_{i}\overset{h(TID_{i},X_{k})}{⇌}GWN}$

  According to $A_{15}$, $D_{22}$ and message meaning rule, we reach:

  $D_{23}$. $U_{i}\left|\equiv GWN\right|∼\left(r_{k}⊕r_{j},r_{i},ID_{{SN}_{j}},ID_{GWN},T_{2},T_{3},T_{4}\right)$

  According to $A_{1}$, $D_{23}$ and freshness distribution rule, we attain:

  $D_{24}$. $U_{i}\left|\equiv GWN\right|\equiv \#\left(r_{k}⊕r_{j},r_{i},ID_{{SN}_{j}},ID_{GWN},T_{2},T_{3},T_{4}\right)$

  According to $D_{23}$-$D_{24}$ and nonce-verification rule, we seek:

  $D_{25}$. $U_{i}\left|\equiv GWN\right|\equiv \left(r_{k}⊕r_{j},r_{i},ID_{{SN}_{j}},ID_{GWN},T_{2},T_{3},T_{4}\right)$

  According to $D_{25}$ and belief rule, we extract:

  $D_{26}$. $U_{i}\left|\equiv GWN\right|\equiv \left(r_{k}⊕r_{j}\right)$

  According to $D_{19}$, $A_{13}$, we get:

  $D_{27}$. $U_{i}|\equiv GWN\Rightarrow \left(r_{k}⊕r_{j}\right)$

  According to $D_{26}$-$D_{27}$, $A_{1}$ and jurisdiction rule, we obtain:

  $D_{28}$. $U_{i}|\equiv r_{k}⊕r_{j}$

  According to $D_{28}$, $A_{1}$ and $SK=h(r_{j}⊕r_{k}⊕r_{j})$, we gain:

  $Aim_{7}$. $U_{i}|\equiv U_{i}\overset{SK}{⇌}GWN$

  According to $K_{i}$, we seek:

  $D_{29}$. $U_{i}◃<SK,T_{4},h\left(TID_{i},X_{k}\right){>}_{GWN\overset{SK}{⇌}U_{i}}$

  According to $D_{29}$, $Aim_{4}$, $Aim_{7}$ and message meaning rule, we obtain:

  $D_{30}$. $U_{i}\left|\equiv GWN\right|∼\left(SK,T_{4},h\left(TID_{i},X_{k}\right)\right)$

  According to $D_{30}$, $Aim_{7}$ and nonce-verification rule, we reach:

  $Aim_{8}$: $U_{i}\left|\equiv GWN\right|\equiv SK$

  According to $Aim_{3}$ and $Aim_{5}$, we ge

  $Aim_{9}$: $SN_{j}\left|\equiv U_{i}\right|\equiv U_{i}\overset{SK}{⇌}SN_{j}$

  According to $Aim_{6}$ and $Aim_{8}$, we get:

  $Aim_{10}$: $U_{i}\left|\equiv SN_{j}\right|\equiv U_{i}\overset{SK}{⇌}SN_{j}$

### 5.3. Formal Security Proof

In order to show that our scheme is secure, we first define the following assumption:

***The encryption algorithm*** Ω ***assumption***: Ω is secure if $Adv_{A}^{\Omega }\leq \epsilon$ for any sufficiently small ε>0, any probabilistic, polynomial time adversary A, where $Adv_{A}^{\Omega }$ denotes the Ω-advantage.

**Theorem 1.**  *Let* Ω *be secure. Under the assumption that the one-way hash function h(·) closely behaves as an oracle, the proposed scheme is provably secure against an adversary for protecting user anonymity and session key.*

We consider the following two random oracles to construct an adversary A:

***Reveal 1***: This oracle will unconditionally output the value *x* from the given hashed result y=h(x).

***Reveal 2***: This oracle will unconditionally output the plaintext *x* from the given ciphertext $C=Enc_{k}\left(x\right)$.

**Proof of Theorem 1.**  We assume that A has the ability to derive the identity $ID_{i}$ of the user $U_{i}$ and the session key SK among $U_{i}$, the gateway node GWN and the sensor node $SN_{j}$. Then he needs to execute the following experimental algorithm, say $EXP1_{A}^{\Omega }$ (Algorithm 1), $EXP2_{A}^{Hash}$ (Algorithm 2) for our proposed scheme. Define the success for $EXP1_{A}^{\Omega }$ as $Succ1_{A}^{\Omega }=Pr\left[EXP2_{A}^{\Omega }=1\right]-1$, $EXP2_{A}^{Hash}$ as $Succ2_{A}^{Hash}=Pr\left[EXP2_{A}^{Hash}=1\right]-1$, and the advantage for $EXP1_{A}^{\Omega }$ becomes $Adv1_{A}^{\Omega }\left(t_{1},q_{1}\right)=max_{A}Succ1_{A}^{\Omega }$, the advantage for $EXP1_{A}^{\Omega }$ becomes $Adv2_{A}^{Hash}\left(t_{2},q_{2}\right)=max_{A}Succ2_{A}^{Hash}$, where $t_{i}$ denotes the maximum time interval, $q_{i}$ denotes the number of queries to the Reveali(i=1,2) oracle. However, according to Ω assumption and the one-way property of hash function, both they are hard problems within polynomial time, *i.e.*, $Adv1_{A}^{\Omega }\left(t_{1},q_{1}\right)\leq \epsilon$, $Adv2_{A}^{Hash}\left(t_{2},q_{2}\right)\leq \epsilon$, for any sufficiently small ϵ>0. As a result, there is no way for the adversary A to retrieve the user identity $ID_{i}$ and the session key SK. ☐

**Algorithm 1**
$EXP1_{A}^{\Omega }$.  1:Eavesdrop the login message $\left\{D_{i},E_{i}\right\}$, $D_{i}=Enc_{h(TID_{i},X_{k})}\left(ID_{i},T_{1},TID_{i},r_{i}\right)$, $E_{i}=h\left(h\left(ID_{i},X_{k}\right),r_{i},T_{1}\right)$  2:Call Reveal1 oracle. Let $\left(ID_{i}^{{}^{\prime }},T_{1}^{{}^{\prime }},TID_{i}^{{}^{\prime }},r_{i}^{{}^{\prime }}\right)\leftarrow Reveal1\left(D_{i}\right)$  3:Intercept the authenticated message $\left\{F_{i},G_{i}\right\}$, where $F_{i}=E_{h(ID_{SN_{j}}⊕S_{ran})}\left(r_{k}⊕r_{i},TID_{i},T_{1},T_{2}\right)$, $G_{i}=h\left(TID_{i},ID_{SN_{j}},h\left(ID_{SN_{j}}⊕S_{ran}\right),ID_{GWN},T_{2},r_{k},r_{i}\right)$.  4:Call Reveal1 oracle. Let $\left(r_{k}^{*},r_{i}^{*},TID_{i}^{*},T_{1}^{*},T_{2}^{*}\right)\leftarrow Reveal\left(F_{i}\right)$  5:**If** ($T_{1}^{\prime }=T_{1}^{*}$) **then**  6:Accept $ID_{i}^{\prime }$ as the true identity of the user $U_{i}$  7:**return 1**  8:**else**  9:**return 0**10:**end if**

**Algorithm 2**
$EXP2_{A}^{Hash}$.  1:Eavesdrop the authenticated message $\left\{G_{i},F_{i}\right\}$, where $G_{i}=h\left(TID_{i},ID_{SN_{j}},h\left(ID_{SN_{j}}⊕S_{ran}\right),ID_{GWN},T_{2},r_{k},r_{i}\right)$, $F_{i}=E_{h(ID_{SN_{j}}⊕S_{ran})}\left(r_{k}⊕r_{i},TID_{i},T_{1},T_{2}\right)$  2:Call Reveal2 oracle. Let $(TID_{i}^{{}^{\prime }},ID_{SN_{j}}^{{}^{\prime }},h(ID_{SN_{j}}⊕$  3:Eavesdrop the communicated message $\left\{I_{i},H_{i}\right\}$, $I_{i}=h\left(ID_{SN_{j}},TID_{i},T_{3},SK\right)$, $H_{i}=Enc_{h(ID_{SN_{j}}⊕S_{ran})}\left(r_{j},T_{3},r_{k},r_{i}\right)$  4:Call Reveal2 oracle. Let $\left(ID_{SN_{j}}^{{}^{\prime \prime }},TID_{i}^{{}^{\prime \prime }},T_{3}^{{}^{\prime \prime }},SK^{{}^{\prime \prime }}\right)\leftarrow Reveal2\left(D_{i}\right)$  5:**If** ($TID_{i}^{{}^{\prime }}=TID_{i}^{{}^{\prime \prime }}$) **then**  6:Accept $SK^{\prime }$ as the session key among $U_{i}$, GWN and $SN_{j}$  7:**return 1**  8:**else**  9:**return 0**10:**end if**

### 5.4. Simulation Results Using AVISPA Tool

AVISPA is one of the publicly accepted Internet schemes verification techniques among many developed semi-automated formal security analysis tools and several schemes [30,31] have been analyzed using it. It is a push-button tool for error detection based on the Dolev and Yao model [32] and provides a modular role-based expressive formal language called the HLPSL (High level protocol specification language) for targeting the design of the schemes. The HLPSL presentation of the protocol is translated into the lower level description language called IF (Intermediate Format) by the translator called HLPSL2IF, which is the entrance of architecture of AVISPA. IF presentation of the scheme is used as the start point to the four various back-ends: OFMC (On the-fly Model-Checker), CL-AtSe (CL-based Attack Searcher), SATMC (SAT-based Model-Checker) and TA4SP (Tree-Automata based Protocol Analyzer). These back-ends are utilized to analyze different security properties such as secrecy of the shared session key, authentication, the privacy of user and robustness against replay attacks. The OF (output format) is generated by using one of the four back-ends which measures whether the security scheme is SAFE or UNSAFE and under what conditions it has been obtained.

**Figure 4:**
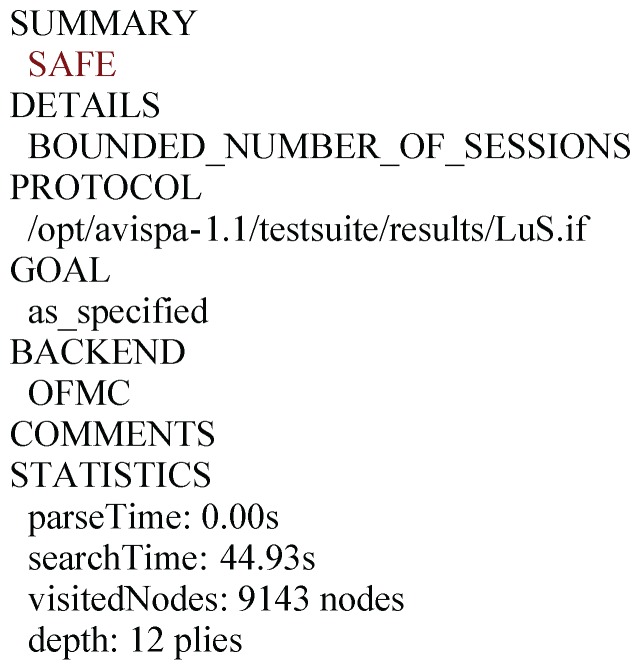
Simulation result for the OFMC.

**Figure 5:**
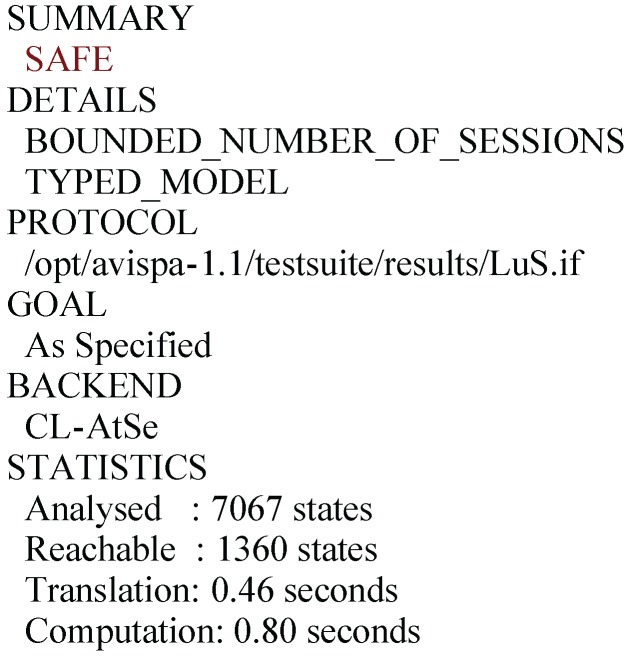
Simulation result for the CL-AtSe.

**Figure A1:**
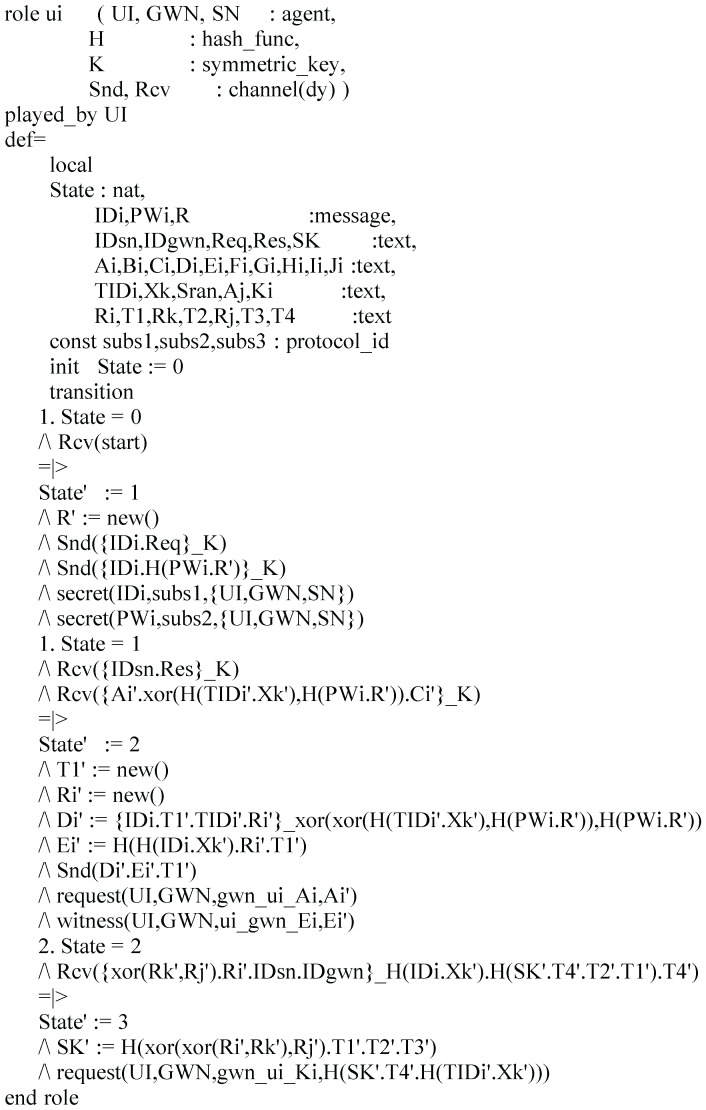
Role specification for the user $U_{i}$.

**Figure A2:**
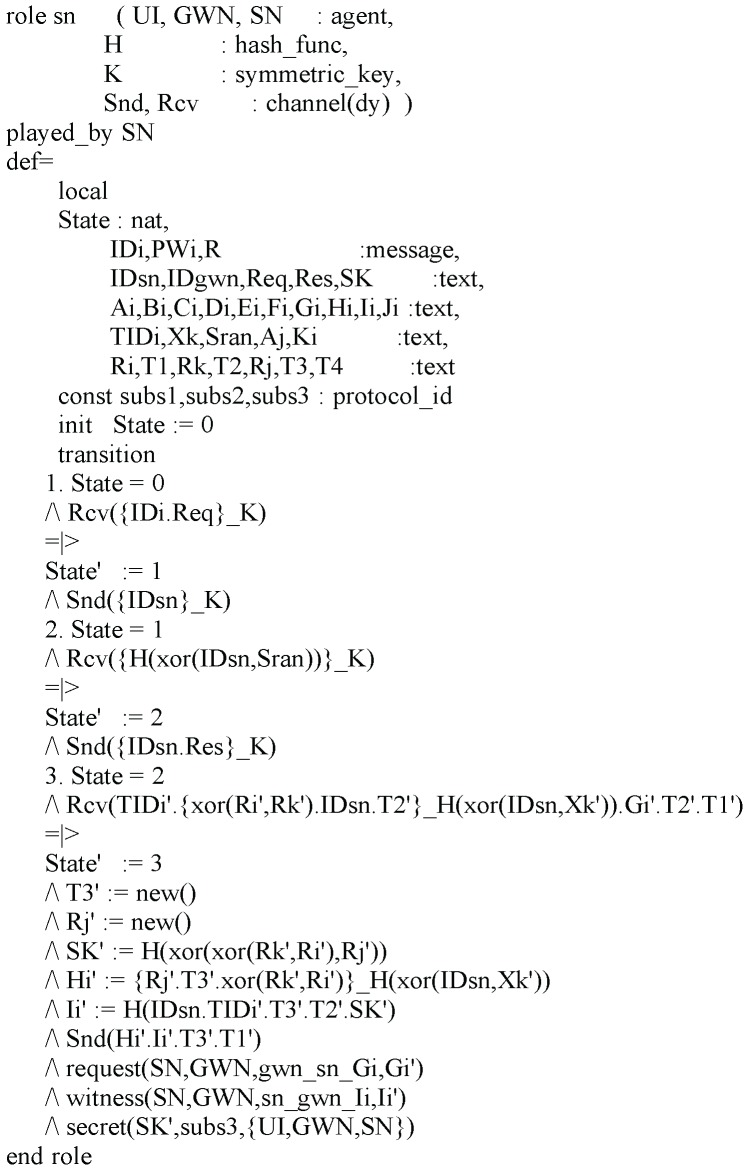
Role specification for the sensor node $SN_{j}$.

**Figure A3:**
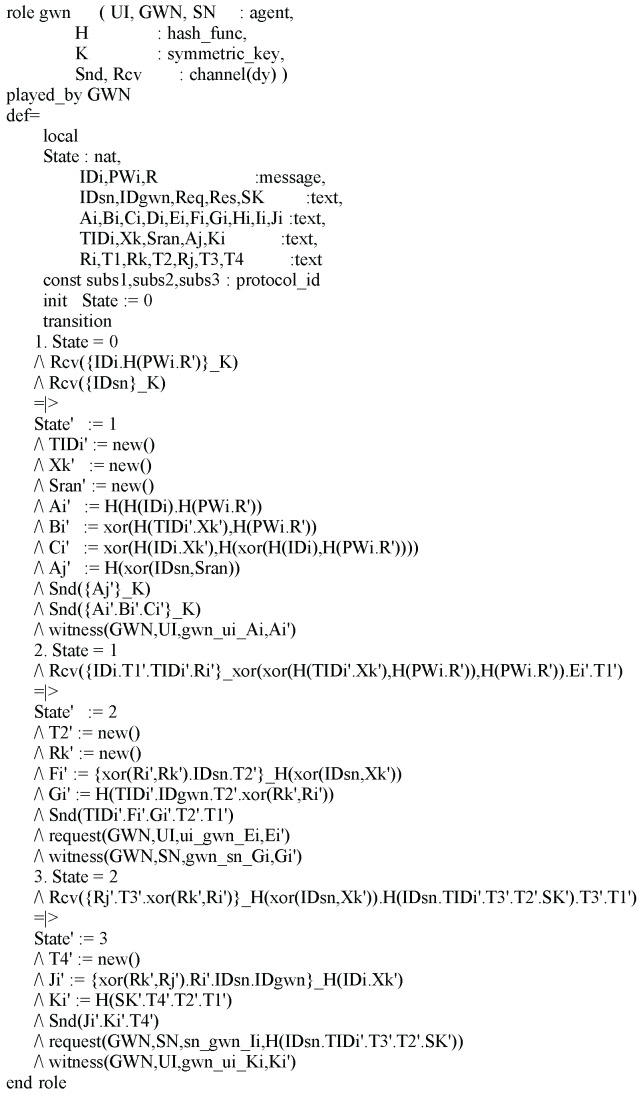
Role specification for the gateway node GWN.

**Figure A4:**
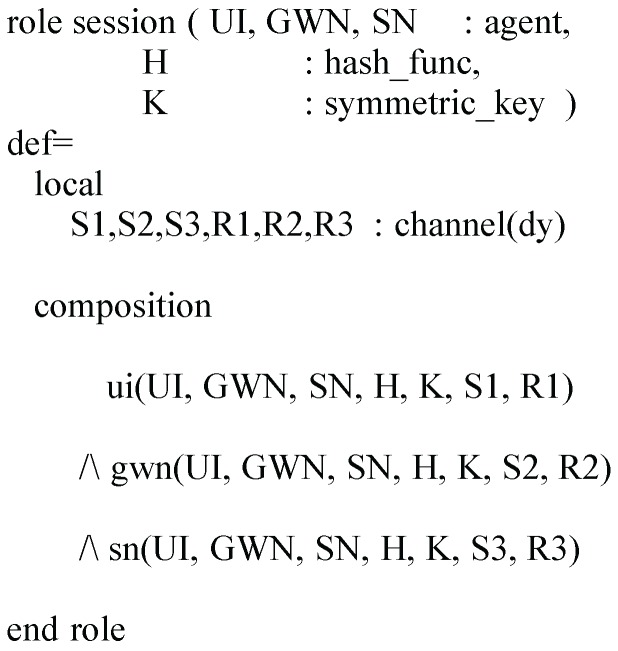
Role specification for the session.

**Figure A5:**
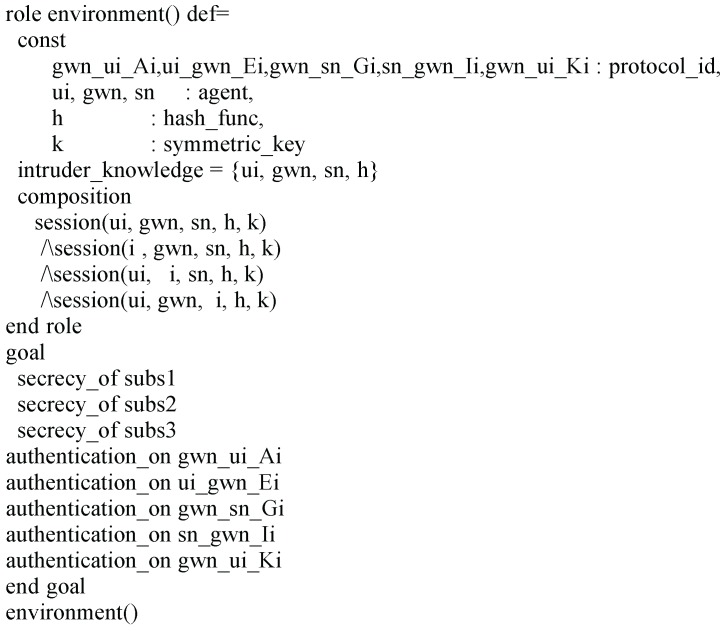
Role specification for the environment.

In order to evaluate the security of the proposed AKA scheme by the AVISPA tools, we have implemented the specifications for the user $U_{i}$ (Appendix A, Figure A1), the sensor node $SN_{j}$ (Appendix A, Figure A2), the gate-way node GWN (Appendix A, Figure A3), the session (Appendix A, Figure A4), goal and the environment (Appendix A, Figure A5) in HLPSL. The desired goals, mutual authentication between $U_{i}$ and GWN by checking $E_{i}$ and $K_{i}$, between GWN and $SN_{j}$ by checking $G_{i}$ and $I_{i}$, the secrecy of session key, user’s identity and password are all achieved. We have chosen the widely-accepted OFMC and CL-AtSe back-ends for the execution tests and a bounded number of sessions model checking. In OFMC backend (Figure 4), the depth for the search is 12, the total number of nodes searched in this case is 9143, which takes 44.93 s. In CL-AtSe backend (Figure 5), 7067 states were analyzed and 1360 states were reachable. Further, CL-AtSe backend took 0.46 s for translation and 0.8 s for computation. After simulation of the code through OFMC and CL-AtSe back-ends, the results show the proposed AKA scheme is guard against both the active and passive adversaries.

## 6. Performance Analysis

This section summarily presents the performance of the proposed AKA scheme and compares in terms of security analysis and computation overheads with existing hash-function based schemes. While computing the cost of the scheme, we assume the length of the identity is 128 bits, the AES encryption/decryption [33] require each 128 bits, the timestamp is 24 bits and the message digest of SHA-3 [34] is 256 bits. Let $T_{h}$ be the time for one hashing operation, and $T_{s}$ be the time for one symmetric cryptography operation, we omit xor operation due to its negligible computational cost.

Table 2 shows the computational complexity and communication overhead analysis along the main security attributes with schemes Aim-Biswas [24], Farash *et al.* [23], Turkanović *et al.* [21] and Xue *et al.* [19] It is noted that the communication parameters of the proposed scheme are $\left\{ID_{i},h\left(PW_{i},r\right),ID_{SN_{j}}\right),A_{i},B_{i},C_{i},A_{j},D_{i},E_{i},F_{i},G_{i},H_{i},I_{i},J_{i},K_{i}\}=128\times 2+256\times 13=3680$ bits, the cost of registration is $9T_{h}$, during the authentication process, the computation cost of the GWN is $5T_{h}+3T_{s}$, the computation cost of the simple resource constrained sensor node is $4T_{h}+2T_{s}$, the total time spent by the proposed scheme is $22T_{h}+7T_{s}$. According to our experiment results using the jPBC library (2.0.0, [35]) (CPU: 3.2 GHz, RAM: 4.0 GB), the arithmetic mean for executing $T_{h}$ is 0.0359 ms, $T_{s}$ is 0.1755 ms after running them 1000 times. Thus, the execution time of the user side is 0.6023 ms, the resource constrained sensor node is 0.4946 ms, the GWN is 0.9214 ms and the total execution time of the proposed AKA scheme is 2.0183 ms. The results shows that the computational cost of the user and the gateway node are considered to be taken on more than sensor node part due to its resource constrained environment. From Table 2, we can see that Farash *et al.*’s scheme [23] achieves more security, that is, resistance to stolen smart card attack and protection of sensor node’s identity, although Farash *et al.*’s scheme consumes more computations than Turkanović *et al.* [21]. Even though the efficiency of Aim-Biswas’s scheme [24] is higher than Turkanović *et al.* [21]’s scheme, Aim-Biswas’s scheme is still vulnerable to known session-specific temporary information attack and no protection of sensor node anonymity. Xue *et al.* [19] is insecure against sensor node impersonation attack and denial-of-service attack excepts vulnerability to known session-specific temporary information attack even though its computational overheads is lower than Farash *et al.*’s scheme. Compared with other four schemes which cannot ensure known session-specific temporary information attack resistance, the proposed AKA scheme consumes a slight higher computation cost lies in using symmetric cryptographic operations. In the face of the perspective of practical application, we consider the security of a cryptographic protocol is the most important. It is acceptable with such high level of security at the expense of increasing computational cost moderately. Therefore, the proposed AKA scheme is very efficient and practical for the resource constrained WSNs environment.

## 7. Conclusions

In this paper, we review and show that Amin-Biswas’s scheme is susceptible to known session-specific temporary information attack, thus suffering from various kinds of attacks, such as user impersonation, off-line password guessing attacks and leakage of user identity. In order to erase the drawbacks of Amin-Biswas’s scheme, we propose an anonymous AKA scheme for WSNs by using the lightweight operations, such as one-way hash functions, xor and symmetric cryptography. The proposed anonymous AKA scheme is characterized to provide relatively more security features and high security level, simulation results confirmed the efficiency of our proposal in terms of the computation and communication overheads. We are interested in extending the integration of biometrics to design a relatively more efficiency AKA scheme without compromising several security aspects in future.

## Figures and Tables

**Table 1:** Notations.

Symbol	Description
$U_{i}$	User
GWN	Gateway node
$SN_{j}$	Sensor node
HGWN	Home gateway node
$ID_{i}/PW_{i}$	Identity/Password of $U_{i}$
$TID_{i}$	Random identity of $U_{i}$ generated by GWN for authentication
$ID_{SN_{j}}$	Identity of $SN_{j}$
$X_{k}$	Secret key of GWN
ΔT	Constant transmission time
$T_{i}$	Timestamp
*r*/$r_{i}$	Random numbers of $U_{i}$
h(·)	One-way hash function
⊕	Xor operation

**Table 2:** Performance analysis.

	Ours	Aim-Biswas [24]	Farash *et al.* [23]	Turkanović *et al.* [21]	Xue *et al.* [19]
Communication cost (bits)	3680	3808	3808	2816	3212
Computation cost (user)	$7T_{h}+2T_{s}$	$9T_{h}$	$13T_{h}$	$9T_{h}$	$8T_{h}$
Computation cost (sensor)	$4T_{h}+2T_{s}$	$5T_{h}$	$11T_{h}$	$6T_{h}$	$8T_{h}$
Computation cost (GWN)	$11T_{h}+3T_{s}$	$11T_{h}$	$23T_{h}$	$12T_{h}$	$18T_{h}$
Total (ms)	2.0183	0.8975	1.6873	0.9693	1.2206
R1	Yes	No	No	No	No
R2	Yes	Yes	Yes	Yes	No
R3	Yes	Yes	Yes	Yes	Yes
R4	Yes	Yes	Yes	Yes	No
R5	Yes	Yes	Yes	Yes	Yes
R6	Yes	Yes	Yes	No	Yes
R7	Yes	No	Yes	No	Yes

R1: Resiliency of known session-specific temporary information attack; R2: Resiliency of denial-of-service attack; R3: Resiliency of insider attack; R4: Resiliency of sensor node impersonation attack; R5: User identity protection; R6: Resiliency of stolen smart card attack; R7: Sensor node anonymity.

## Appendix A HLPSL Implementation of the Proposed Scheme

This section shows the proposed AKA for the roles of the user $U_{i}$ (Figure A1), the gateway node GWN (Figure A2), the sensor node $SN_{j}$ (Figure A3), the session (Figure A4) and the environment (Figure A5).
